# Brain structure and connectivity mediate the association between lifestyle and cognition: The Maastricht Study

**DOI:** 10.1093/braincomms/fcae171

**Published:** 2024-05-16

**Authors:** Nathan R DeJong, Jacobus F A Jansen, Martin P J van Boxtel, Miranda T Schram, Coen D A Stehouwer, Marleen M J van Greevenbroek, Carla J H van der Kallen, Annemarie Koster, Simone J P M Eussen, Bastiaan E de Galan, Walter H Backes, Sebastian Köhler

**Affiliations:** Faculty of Health, Medicine and Life Sciences, School for Mental Health & Neuroscience, Maastricht University, 6229 ER Maastricht, The Netherlands; Department of Psychiatry and Neuropsychology, Faculty of Health, Medicine and Life Sciences, Maastricht University, 6229 ER Maastricht, The Netherlands; Alzheimer Centrum Limburg, Maastricht University Medical Center+, 6229 ET Maastricht, The Netherlands; Department of Radiology & Nuclear Medicine, Maastricht University Medical Center+, 6229 HX Maastricht, The Netherlands; Faculty of Health, Medicine and Life Sciences, School for Mental Health & Neuroscience, Maastricht University, 6229 ER Maastricht, The Netherlands; Department of Radiology & Nuclear Medicine, Maastricht University Medical Center+, 6229 HX Maastricht, The Netherlands; Department of Electrical Engineering, Eindhoven University of Technology, 5612 AP Eindhoven, The Netherlands; Faculty of Health, Medicine and Life Sciences, School for Mental Health & Neuroscience, Maastricht University, 6229 ER Maastricht, The Netherlands; Alzheimer Centrum Limburg, Maastricht University Medical Center+, 6229 ET Maastricht, The Netherlands; Department of Radiology & Nuclear Medicine, Maastricht University Medical Center+, 6229 HX Maastricht, The Netherlands; Faculty of Health, Medicine and Life Sciences, School for Mental Health & Neuroscience, Maastricht University, 6229 ER Maastricht, The Netherlands; Faculty of Health, Medicine and Life Sciences, School for Cardiovascular Diseases (CARIM), Maastricht University, 6229 ER Maastricht, The Netherlands; Department of Internal Medicine, Maastricht University Medical Center+, 6229 HX Maastricht, The Netherlands; Maastricht Heart & Vascular Center, Maastricht University Medical Center+, 6229 HX Maastricht, The Netherlands; Faculty of Health, Medicine and Life Sciences, School for Cardiovascular Diseases (CARIM), Maastricht University, 6229 ER Maastricht, The Netherlands; Department of Internal Medicine, Maastricht University Medical Center+, 6229 HX Maastricht, The Netherlands; Faculty of Health, Medicine and Life Sciences, School for Cardiovascular Diseases (CARIM), Maastricht University, 6229 ER Maastricht, The Netherlands; Department of Internal Medicine, Maastricht University Medical Center+, 6229 HX Maastricht, The Netherlands; Faculty of Health, Medicine and Life Sciences, School for Cardiovascular Diseases (CARIM), Maastricht University, 6229 ER Maastricht, The Netherlands; Department of Internal Medicine, Maastricht University Medical Center+, 6229 HX Maastricht, The Netherlands; Faculty of Health, Medicine and Life Sciences, Care and Public Health Research Institute (CAPHRI), Maastricht University, 6229 ER Maastricht, The Netherlands; Department of Social Medicine, Faculty of Health, Medicine and Life Sciences, Maastricht University, 6229 GT Maastricht, The Netherlands; Faculty of Health, Medicine and Life Sciences, School for Cardiovascular Diseases (CARIM), Maastricht University, 6229 ER Maastricht, The Netherlands; Faculty of Health, Medicine and Life Sciences, Care and Public Health Research Institute (CAPHRI), Maastricht University, 6229 ER Maastricht, The Netherlands; Department of Epidemiology, Maastricht University Medical Center+, 6229 HX Maastricht, The Netherlands; Faculty of Health, Medicine and Life Sciences, School for Cardiovascular Diseases (CARIM), Maastricht University, 6229 ER Maastricht, The Netherlands; Department of Internal Medicine, Maastricht University Medical Center+, 6229 HX Maastricht, The Netherlands; Department of Internal Medicine, Radboud University Medical Centre, 6500 HB Nijmegen, The Netherlands; Faculty of Health, Medicine and Life Sciences, School for Mental Health & Neuroscience, Maastricht University, 6229 ER Maastricht, The Netherlands; Department of Radiology & Nuclear Medicine, Maastricht University Medical Center+, 6229 HX Maastricht, The Netherlands; Faculty of Health, Medicine and Life Sciences, School for Cardiovascular Diseases (CARIM), Maastricht University, 6229 ER Maastricht, The Netherlands; Faculty of Health, Medicine and Life Sciences, School for Mental Health & Neuroscience, Maastricht University, 6229 ER Maastricht, The Netherlands; Department of Psychiatry and Neuropsychology, Faculty of Health, Medicine and Life Sciences, Maastricht University, 6229 ER Maastricht, The Netherlands; Alzheimer Centrum Limburg, Maastricht University Medical Center+, 6229 ET Maastricht, The Netherlands

**Keywords:** brain reserve, connectivity, resilience, lifestyle, cognition

## Abstract

Life-course exposure to risk and protective factors impacts brain macro- and micro-structure, which in turn affects cognition. The concept of brain-age gap assesses brain health by comparing an individual’s neuroimaging-based predicted age with their calendar age. A higher BAG implies accelerated brain ageing and is expected to be associated with worse cognition. In this study, we comprehensively modelled mutual associations between brain health and lifestyle factors, brain age and cognition in a large, middle-aged population. For this study, cognitive test scores, lifestyle and 3T MRI data for *n* = 4881 participants [mean age (± SD) = 59.2 (±8.6), 50.1% male] were available from The Maastricht Study, a population-based cohort study with extensive phenotyping. Whole-brain volumes (grey matter, cerebrospinal fluid and white matter hyperintensity), cerebral microbleeds and structural white matter connectivity were calculated. Lifestyle factors were combined into an adapted LIfestyle for BRAin health weighted sum score, with higher score indicating greater dementia risk. Cognition was calculated by averaging *z*-scores across three cognitive domains (memory, information processing speed and executive function and attention). Brain-age gap was calculated by comparing calendar age to predictions from a neuroimaging-based multivariable regression model. Paths between LIfestyle for BRAin health tertiles, brain-age gap and cognitive function were tested using linear regression and structural equation modelling, adjusting for sociodemographic and clinical confounders. The results show that cerebrospinal fluid, grey matter, white matter hyperintensity and cerebral microbleeds best predicted brain-age gap (*R*^2^ = 0.455, root mean squared error = 6.44). In regression analysis, higher LIfestyle for BRAin health scores (greater dementia risk) were associated with higher brain-age gap (standardized regression coefficient *β* = 0.126, *P* < 0.001) and worse cognition (*β* = −0.046, *P* = 0.013), while higher brain-age gap was associated with worse cognition (*β*=−0.163, *P* < 0.001). In mediation analysis, 24.7% of the total difference in cognition between the highest and lowest LIfestyle for BRAin health tertile was mediated by brain-age gap (*β*_indirect_ = −0.049, *P* < 0.001; *β*_total_ = −0.198, *P* < 0.001) and an additional 3.8% was mediated via connectivity (*β*_indirect_ = −0.006, *P* < 0.001; *β*_total_ = −0.150, *P* < 0.001). Findings suggest that associations between health- and lifestyle-based risk/protective factors (LIfestyle for BRAin health) and cognition can be partially explained by structural brain health markers (brain-age gap) and white matter connectivity markers. Lifestyle interventions targeted at high-risk individuals in mid-to-late life may be effective in promoting and preserving cognitive function in the general public.

## Teaser

DeJong *et al*. explore relationships between brain age, cognitive function and lifestyle factors associated with dementia risk in 4881 participants. Brain age was estimated using whole brain and white matter hyperintensity volumes. Linear structural equation modelling found that brain age partially mediates the association between lifestyle factors and cognitive function.

## Graphical Abstract

**Graphical Abstract:**
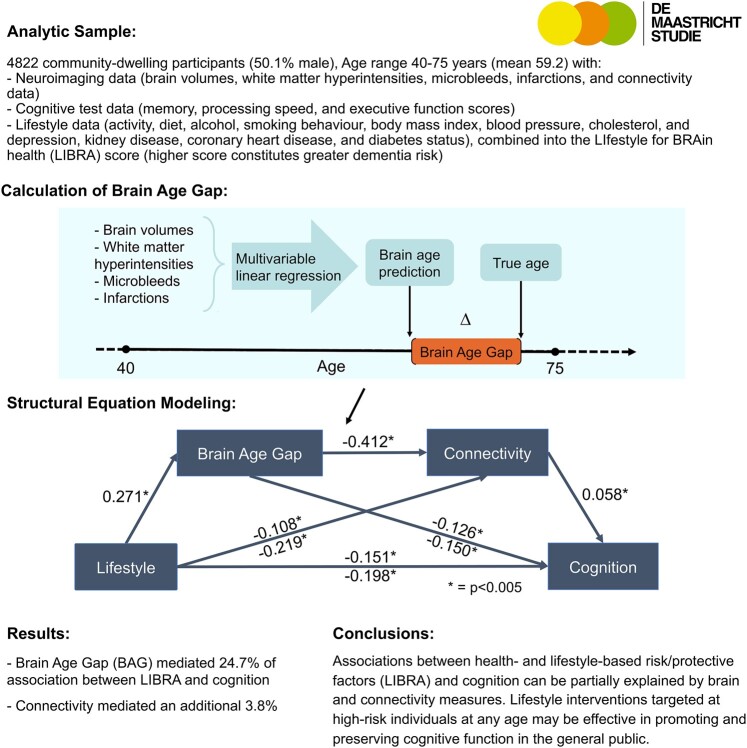

## Introduction

Dementia is a global public health problem affecting an estimated 55 million people worldwide.^1^ It is not a single disease but rather a syndrome, characterized by cognitive decline, and associated with advanced age- or pathology-related organ damage, such as atrophy,^2^ white matter (WM) lesions,^3^ cerebral microbleeds (CMB)^4^ and reduced white matter connectivity.^5^ While effective and broadly indicated pharmacological treatments for dementia are still lacking, in recent years there has been an increased focus on primary prevention of dementia through lifestyle change.^6^ Several modifiable lifestyle factors, such as physical and cognitive activity, healthy diet and refraining from smoking, have been found to be associated with a reduced risk of both brain damage and cognitive decline.^7^

One means of measuring generalized damage to the brain is brain-age gap (BAG) estimation. This technique assesses brain features in relation to a normative age-curve for brain structure, resulting in an individual’s predicted ‘brain age’. Comparison of predicted brain age with one’s true age gives the ‘brain-age gap’, a measure of an individual’s deviation from the normal ageing trajectory, with a positive BAG indicating evidence of premature brain ageing and vice versa. Previous studies on regression-based or machine-learning-based modelling of BAG have demonstrated that a higher BAG is associated with cardiometabolic risk factors,^8^ cognitive decline^9^ and an increased likelihood of conversion from mild cognitive impairment to Alzheimer’s Disease.^10^ However, studies have generally been limited to clinical populations, and have primarily focused on early detection of dementia.

Only a few studies examined whether modifiable dementia risk factors are related with a narrowing or even an inversion of the BAG towards a younger-than expected brain. In one such study, years of education, a characteristic that is considered modifiable at the population level,^6^ was found to be associated with lower BAG.^11^ In another larger study, no significant association between lifestyle (alcohol consumption, smoking behaviour and physical activity level) and BAG was found.^12^

In the present study, we modelled associations between lifestyle factors, brain age and cognition in a large, middle-aged population. We first estimated brain age using whole-brain measures, a technique that has the advantage of low computational requirements and uses data that are readily available in many datasets. We subsequently employed structural equation modelling to assess the contributions of lifestyle factors and the calculated BAG to cognitive function.

## Materials and methods

### Population study design

Data were used from The Maastricht Study, an observational, prospective, population-based cohort study. The rationale and methodology have been described previously.^13^ In brief, the study focuses on the aetiology, pathophysiology, complications and comorbidities of type 2 diabetes mellitus (T2DM) and is characterized by an extensive phenotyping approach. Eligible for participation were all individuals aged between 40 and 75 years and living in the southern part of the Netherlands. Participants were recruited through mass media campaigns and from the municipal registries and the regional Diabetes Patient Registry via mailings. Recruitment was stratified according to known T2DM status with an oversampling of individuals with T2DM for statistical efficiency. The present report includes cross-sectional data from the first 7689 participants, who completed the baseline survey between November 2010 and December 2017. The examinations of each participant were on average performed within a time window of 3 months. Structural and diffusion MRI (dMRI) measurements were implemented from December 2013 onwards until February 2017 and were available in 5241 out of the 7689 participants. Of the 5241 participants with MRI measurements available, 4881 participants had a complete dataset. The study has been approved by the institutional medical ethical committee (NL31329.068.10) and the Dutch Ministry of Health, Welfare and Sports of the Netherlands (Permit 131088-105234-PG). All participants gave their written informed consent.

### MRI assessment

For each participant, MRI data were acquired on a 3T clinical magnetic resonance scanner (MAGNETOM Prismafit, Siemens Healthineers GmbH, Munich, Germany) located at a dedicated scanning facility (Scannexus, Maastricht, the Netherlands) using a head/neck coil with 64 elements for parallel imaging. The MRI protocol included a three-dimensional T1-weighted magnetization-prepared rapid-acquisition gradient-echo sequence [repetition time/inversion time/echo time (TR/TI/TE) 2300/900/2.98 ms, 176 slices, 256 × 240 matrix size, 1.0 mm cubic reconstructed voxel size]; a fluid-attenuated inversion recovery (FLAIR) sequence (TR/TI/TE 5000/1800/394 ms, 176 slices, 512 × 512 matrix size, 0.49 × 0.49 × 1.0 mm reconstructed voxel size); and a dMRI using a spin-echo echo-planar imaging sequence (TR/TE 6100/57 ms, 65 slices, 100 × 100 matrix size, 64 diffusion sensitizing gradient directions (*b* = 1200 s/mm^2^), 2.0 mm cubic reconstructed voxel size) with three additional minimally diffusion-weighted images (*b* = 0 s/mm^2^). Contraindications for MRI assessment included the presence of a non-compatible implant or device, and conditions such as epilepsy or claustrophobia, or pregnancy.

Volumetric measurements [cerebrospinal fluid (CSF), WM, grey matter (GM) and white matter hyperintensity (WMH) volume, in mL] were obtained from T1-weighted and T2-weighted FLAIR images by use of an ISO13485:2012-certified, automated method (which included visual inspection)^14,15^ and were summed to derive total intra-cranial volume. WMH volume was further log transformed to mitigate skewness, and all volumetric measurements were standardized. Cerebral lacunar infarcts and CMB were manually counted by three neuroradiologists in accordance with the Microbleed Anatomical Rating Scale.^16^

Structural connectivity measures were derived from MRI as previously described.^17^ Briefly, the images were spatially registered and segmented into 94 regions according to the automatic anatomical labelling atlas.^18^ White matter tractography was calculated for each participant using a constrained spherical deconvolution-based deterministic tractography algorithm^19^ with 2-mm seed point resolution, randomly placed throughout the brain, step size 1 mm and maximum harmonic degree of 8.^20^ Inter-regional connections were calculated using the Brain Connectivity Toolbox^21^ in MATLAB Release 2016a (The MathWorks Inc., Natick, Massachusetts, USA). Connections consisting of fewer than two tracts were removed to reduce noise. The number of connections for each region (node degree) was calculated and averaged across the whole brain to give average node degree, a measure of white matter connectivity for each individual.

### Cognitive assessments

Cognitive performance was assessed by means of a concise neuropsychological test battery (30 mins).^13^ Cognitive domains were evaluated as follows: memory was evaluated with the Verbal Learning Test for immediate and delayed recall; information processing speed was evaluated with the Stroop Colour-Word Test Part I and II, Concept Shifting Test Part A and B and Letter-Digit Substitution Test; executive function and attention were evaluated with the Stroop Colour-Word Test Part III and Concept Shifting Test Part C.^22-25^ Raw scores for each domain were standardized into *z*-scores with mean of zero and standard deviation of one, with higher scores indicating better performance. For statistical efficiency, a composite cognition score was calculated by averaging the *z*-scores across the three cognitive domains: memory, information processing speed and executive function and attention.

### Lifestyle factors

Demographic and clinical data (BMI, waist circumference, office blood pressure and plasma lipid profile) were measured at baseline. The presence of T2DM was determined by an oral glucose tolerance test after an overnight fast. Hypertension was defined as an office systolic blood pressure >140 mmHg, diastolic blood pressure >90 mmHg or the use of anti-hypertensive medication. Educational level (low, medium and high), history of coronary heart disease, smoking status (never, current and former), physical activity (hours per week, classified by intensity level), alcohol consumption (none, low and high) and adherence to the Dutch Healthy Diet 2015 guidelines^26^ were assessed by questionnaires. The presence of depression was assessed using the Mini International Neuropsychiatric Interview^27^ and the Patient Health Questionnaire (range 0–27, cut-off ≥10).^28^ Chronic kidney disease status was derived from average urinary albumin excretion or Chronic Kidney Disease Epidemiology Collaboration (CKD-EPI) equation estimated glomerular filtration rate using serum creatinine (rate of <60 ml/min/1.73 m2).

**Figure 1:**
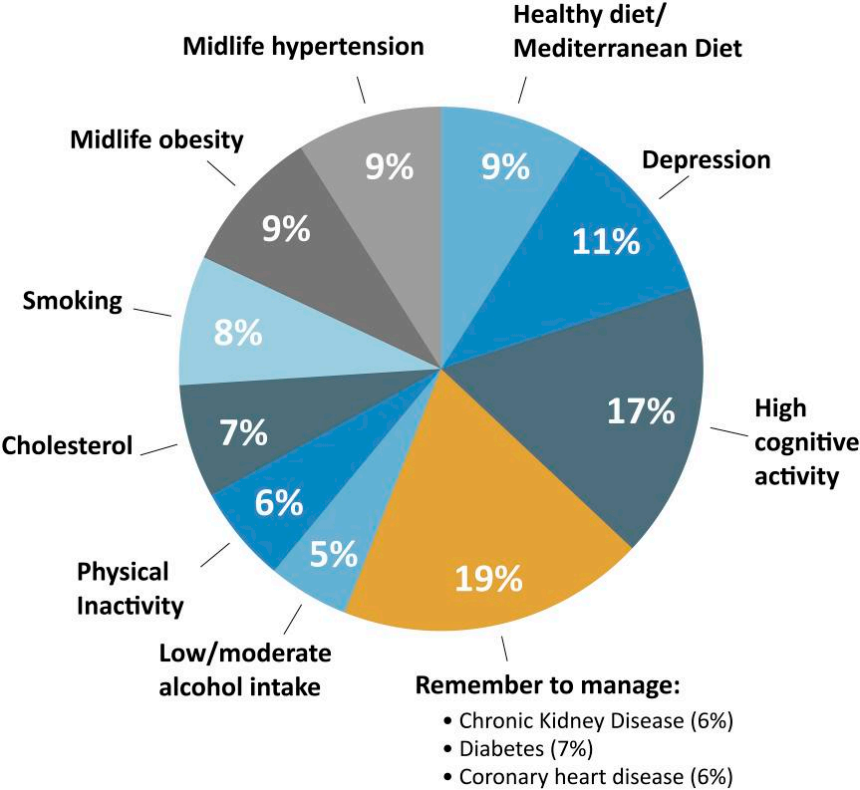
**LIBRA composition.** A summary of the weighted modifiable health- and lifestyle-based risk and protective factors that are combined in the LIBRA score. All factors except for cognitive activity were available from the Maastricht Study.

These factors were combined into an adapted LIBRA score, which provides an individual’s potential for dementia-risk reduction based on a weighted sum score of 12 modifiable risk and preventive factors. Factor selection was based on a systematic literature review and Delphi consensus study,^29^ and weighted based on relative risks for each factor extracted from meta-analysis (see Fig. 1). The LIBRA score has been validated in different studies for prediction of cognitive decline, dementia, brain damage and treatment effect in intervention trials to promote a brain-healthy lifestyle.^7,30-34^

LIBRA score was calculated for each individual from baseline measurements as described previously.^7^ Briefly, measurements for each LIBRA factor were dichotomized and weighted as presented in Table 1. The available factors were then combined into a single weighted sum score (Fig. 1) such that a higher score constitutes greater dementia risk. The level of occupational or leisure time cognitive activity was not available in the dataset, so this LIBRA factor was omitted from the present operationalization. In subsequent analyses, the LIBRA score was found to have a non-linear association with the combined cognition score, and was therefore split into tertiles to allow for linear structural equation modelling.

**Table 1:** Operationalization of LIBRA factors

LIBRA factor	Weight	Operationalization in the Maastricht Study
Adherence to Mediterranean diet	−1.7	Greek Mediterranean diet score (range 0–9) based on a 253-item Food Frequency Questionnaire (FFQ; 1 year reference period). Scores of ≥ 6 are categorized as ‘adherence to the diet’.
Low-to-moderate alcohol intake	−1.0	Self-reported alcohol intake based on the FFQ. Low-to-moderate alcohol use was defined as <70 g/week.
Physical inactivity	+1.1	<150 min/week of (self-reported on CHAMPS questionnaire) moderate-to-vigorous physical activity in the past 2 weeks was categorized as ‘physically inactive’.
Smoking	+1.5	Self-reported data on smoking cigarettes based on an item of the FFQ.
Obesity	+1.6	BMI ≥ 30 kg/m^2^ calculated from physical examination.
Hypertension	+1.6	Average systolic blood pressure ≥ 140 mmHg, or diastolic blood pressure ≥ 90, and/or current anti-hypertensive medication use.
High cholesterol	+1.4	Serum total cholesterol ≥ 6.5 mmol/l.
Type-2 diabetes	+1.3	Glucose tolerance status based on fasting glucose (≥ 7.0) or oral glucose tolerance test (≥ 11.1), or information on current diabetes medications.
Heart disease	+1.0	Self-reported history of cardiovascular disease (cerebrovascular accidents excluded).
Chronic kidney disease	+1.1	Levels of serum cystatin C of < 60 and/or average albuminuria categories, based on average urinary albumin excretion. Microalbuminuria and macroalbuminuria were defined as risk.
Depression	+2.1	Current major or minor depressive episode based on the MINI or presence of moderate-to-severe depressive symptoms based on the PHQ9 (range 0–27; cut-off ≥ 10).
Cognitive activity	−3.2	Not assessed in dataset

LIBRA, LIfestyle for BRAin health index; BMI, body mass index; MINI, mini international neuropsychiatric interview; PHQ9, Patient Health Questionnaire.

### Statistical analyses

First, brain-age modelling was performed by multivariable linear regression with age as a dependent variable and factors of brain integrity as independent variables, including vascular damage (WMH volume, number of CMB and infarctions), brain volumes (GM, white matter and CSF volume) and structural connectivity. These factors were ranked based on the strength (standardized *β*) and significance (*P* ≤ 0.05) of their association with age, as tested by separate linear regression analyses. The presence of non-linear associations were also tested for, based on observations in the previous literature that brain features do not always change linearly with age.^35^

According to this ranking, the most relevant factors from each category (volumetric measures, vascular measures and structural connectivity) were then combined in a single multivariable regression model. Non-significant measures were discarded, resulting in Model 1. Subsequently, the next relevant features were added in a step-wise fashion to the model, resulting in Model 2. Finally, the interactions between features were also added to the model. This results in a model with many correlated predictors, therefore, an automated Least Absolute Shrinkage and Selection Operator (LASSO) regression analysis^36^ was used for model selection in the third and final model. LASSO is a feature selection and regularization technique designed for use in linear regression models with many correlated predictors, which encourages simple, sparse models by incorporating a penalty equal to the absolute magnitude of the coefficient. In this way, small coefficient values are eliminated from the model. The performance of the three models was assessed by comparing *R*^2^ and root mean square error values, and the most parsimonious model was selected. Finally, each individual’s BAG was calculated by subtracting the model-predicted age from true age.

### Path analyses

The associations between BAG and cognitive function, and LIBRA and BAG, were tested using linear regression analysis, controlling for age, sex, education and diabetes status (to account for sampling strategy). Finally, a mediation analysis was performed using structural equation modelling to determine the extent to which BAG mediates the association between LIBRA and cognition. Because of the curvilinear association between LIBRA and cognition, for this analysis LIBRA was binned into tertiles and tested as a categorical rather than continuous variable. The total, direct and indirect (mediated) effects associated with each path were estimated by effect decomposition. All analyses were performed in Stata 17.0. For all analyses, *P* < 0.05 was considered statistically significant.

## Results

### Study population

**Figure 2:**
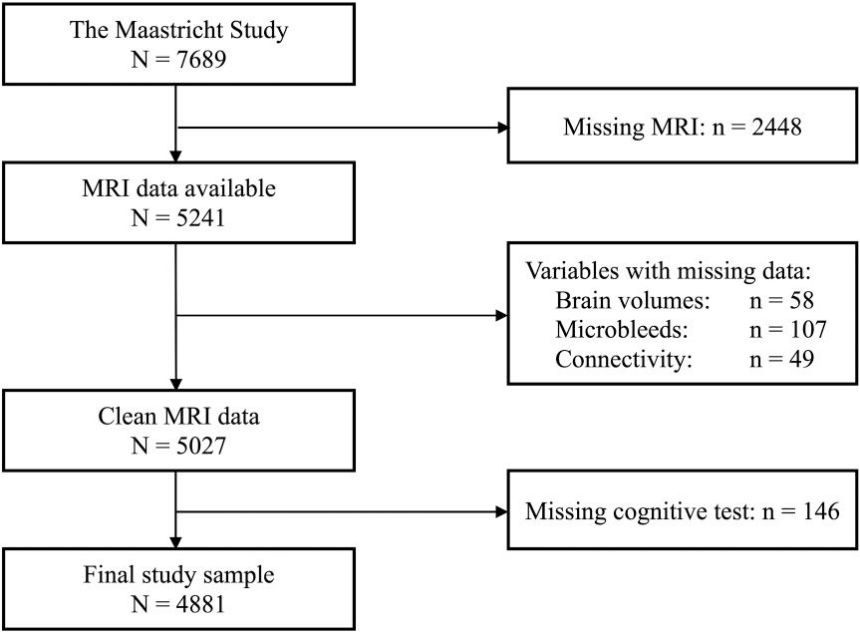
**Participant flowchart.** Sample flowchart, depicting which variables had missing data, resulting in the final analytic sample *N* = 4881.

In total, 4881 participants were included in the present analyses (Fig. 2). Relative to the total population of The Maastricht Study, the participants were younger, less likely to be male and had better cognitive function on average (Table 2).

**Table 2:** Study population characteristics

	Overall	LIBRA score = low	LIBRA score = medium	LIBRA score = high
Number of participants	4881	1628	1710	1543
Age	60.5 (8.7)	58.1 (8.8)	60.6 (8.5)	62.9 (8.2)
Sex (% male)	50.1	44.0	48.7	58.1
Level of education				
% low	31.3	21.2	30.1	43.3
% medium	28.6	29.3	29.7	26.7
% high	40.2	49.5	40.3	30.0
Glucose metabolism status				
% normal	64.9	87.1	66.3	39.8
% pre-diabetes	14.7	11.2	16.2	16.5
% type 2 diabetes	19.9	1.2	16.6	43.4
% other diabetes	0.6	0.4	0.9	0.3
Heart disease	10.0	3.8	6.8	20.1
Previous stroke	2.7	1.3	2.5	8.3
Depression	3.2	0.3	1.4	4.1
Brain age	60.5 (5.9)	59.7 (4.3)	60.4 (4.3)	61.5 (4.3)
Brain-age gap	0.0 (6.4)	−0.8 (4.3)	−0.1 (4.3)	1.0 (4.3)
Cognition score	0.5 (0.7)	0.3 (0.6)	0.1 (0.7)	−0.2 (0.7)
LIBRA score	1.2 (2.0)	−0.9 (0.8)	1.1 (0.6)	3.6 (1.2)

Participant characteristics presented as % or mean (SD) for the whole population, and additionally for each tertile of the Lifestyle for BRAin health (LIBRA) score. Means of brain age and BAG are estimated marginal means per LIBRA tertile, adjusted for age.

### Brain-age model

The performance of the different brain-age prediction models is given in Table 3. While each predictor was individually significant, when combined in multivariable regression, structural connectivity and presence of lacunar infarctions did not contribute additional information significantly related to age. Therefore, they were not included in Models 1 and 2. However, due to the possibility of relevant interactions between features, they were included as interaction terms in the LASSO regression model. This model (Model 3) showed only very modest improvement over the less complex Model 2. In the interests of reducing model complexity while still achieving the most parsimonious model, Model 2 was selected as the main model for future analyses. This model is predicted by CSF volume, CSF volume^2^, GM volume, WMH volume, WMH volume^2^ and a dichotomous measure indicating the presence of CMB. While connectivity was not found to have a large contribution to brain-age prediction, it has previously been found to be an important factor in maintaining cognition.^7^ We therefore included it as an individual variable in later structural equation modelling analysis.

**Table 3:** Performance of different brain-age prediction models

	Components	*R* ^2^	RMSE (years)	*P*-value
Model 1	CSF, CSF^2^, WMH, WMH^2^	0.448	6.49	<0.001
Model 2	CSF, CSF^2^, GM, WMH, WMH^2^, CMB	0.455	6.44	<0.001
Model 3	CSF, CSF^2^, GM, WMH, WMH^2^, CMB, Connectivity	0.460	6.41	<0.001

*R* ^2^ fit, root mean square error (RMSE) and *P*-value for each model. Models 1 and 2 are multivariable linear regression models with manual variable selection (model-driven). Model 3 is a data-driven model, selected using LASSO regression.

**Figure 3:**
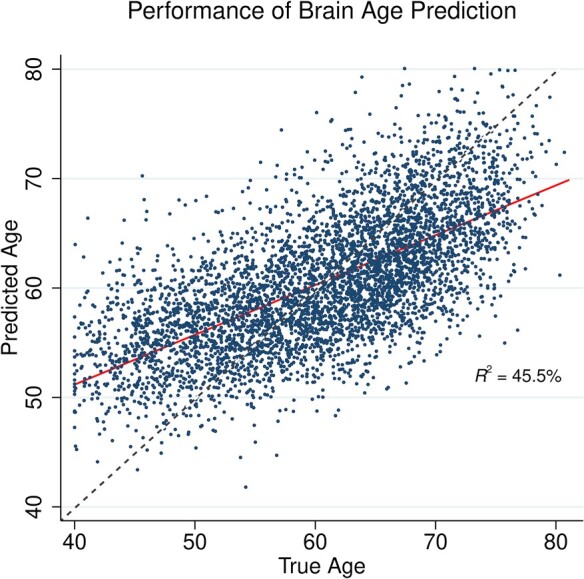
**Brain-age prediction.** Predicted age plotted against true age for each individual (*N* = 4881). Linear best-fit line was calculated (solid line), and compared with the ideal-model-fit line, predicted age = true age (dashed line).

In Fig. 3, the model predictions are plotted against the true age of the participants. Note that due to ‘regression to the mean’, younger individuals are systematically predicted to have higher brain ages, and older individuals, younger brain ages. Therefore, subsequent analyses incorporating brain age were adjusted for age.^37^ Next, each individual’s predicted brain age based on Model 2 was saved and used to calculate BAG as the differences between the predicted brain age and actual age. BAG served as an individual, image-based estimate of brain reserve, with higher BAG representing an ‘older-looking brain’.

### Brain-age associations with lifestyle and cognition

We then separately tested associations between the LIBRA score, BAG and cognition using multiple linear regression. This showed that higher LIBRA scores (higher dementia risk) were associated with higher BAG (standardized regression coefficient *β* = 0.126, *P* < 0.001). A higher BAG was in turn associated with worse cognitive function (*β* = −0.163, *P* < 0.001). In addition, higher LIBRA scores were found to be curvilinearly associated with worse cognitive function (*β*_linear_ = −0.046, *P* = 0.013, *β*_quadratic_ = −0.042, *P* = 0.020).

### Mediation analysis

**Figure 4:**
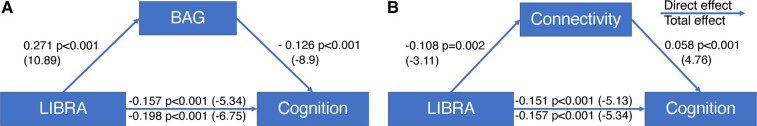
**Mediation of BAG and connectivity on the association between lifestyle and cognition.** Mediation model (*N* = 4881) showing associations between lifestyle and cognitive performance across the highest and lowest tertiles of the LIBRA score, and the PM by (**A**) BAG and (**B**) connectivity, controlling for age, sex, education and time elapsed between imaging and cognitive assessment. Associations are given in standardized coefficients, *z*-statistic in parenthesis.

Next, we performed a mediation analysis, using structural equation modelling (Fig. 4) to test whether the brain maintenance factors summarized by LIBRA are related to better cognition via their association with lower BAG (or better brain reserve). As mentioned previously, for this analysis the sample was split into tertiles according to the LIBRA score. Individuals in the lowest third of LIBRA score (range −2.7–0) were used as reference for those in the highest third of LIBRA score (range 2.0–10.2). This approach was used to maximize the available sample size while also testing dose–response relationships between lifestyle and cognitive function. The total effect of LIBRA on cognition when comparing the lowest and highest tertiles was *β* = −0.198 (*P* < 0.001), which could be decomposed into its indirect effect via BAG on cognition (*β*_indirect_ = −0.041, *P* < 0.001) and the residual direct effect (*β*_direct_ = −0.157, *P* < 0.001), congruent with a partially mediated effect of 20.5% (−0.041/−0.198 = 0.205).

### Additional analyses with structural connectivity

As an additional analysis, we tested the mediation of node degree on the association between LIBRA and cognition, while controlling for the effects of BAG. This resulted in a further mediation of 3.8% (*β*_indirect_ = −0.006, *P* < 0.001; *β*_total_ = −0.157, *P* < 0.001).

**Figure 5:**
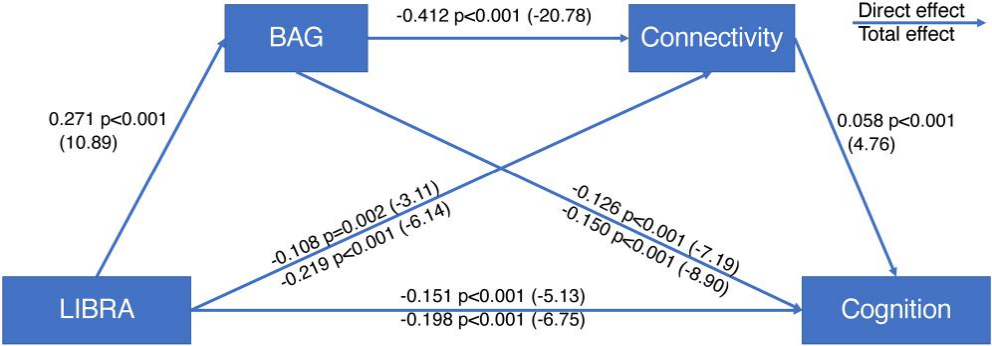
**Combined mediation model.** Mediation model (*N* = 4881) showing associations between lifestyle and cognitive performance across the highest and lowest tertiles of the LIBRA score, and the combined mediation by BAG and connectivity, controlling for age, sex, education and time elapsed between imaging and cognitive assessment. Associations are given in standardized coefficients, *z*-statistic in parenthesis.

We then included both BAG and node degree in a combined mediation analysis, as shown in Fig. 5. This combined model resulted in three mediating pathways for the association of LIBRA and cognition: via BAG alone, via connectivity alone, or via both BAG and connectivity. In this analysis, BAG mediated 17.2% (*β* = −0.034, *P* < 0.001) of the association between LIBRA and cognition, connectivity mediated an additional 3.2% (*β* = −0.006, *P* < 0.001) and the combined pathway mediated an additional 3.3% (*β* = −0.006, *P* < 0.001), resulting in a total mediation of 23.7% (*β*_indirect_ = −0.047, *P* < 0.001; *β*_total_ = −0.157, *P* < 0.001). Additionally, BAG was strongly associated with differences in brain connectivity, and this association mediated ∼16% (*β*_indirect_ = −0.024, *P* < 0.001; *β*_total_ = −0.150, *P* < 0.001) of the association between BAG and cognition.

### Exploratory analyses by cognitive domain

Finally, to better understand which aspects of the combined cognitive score are most associated with measures of lifestyle, BAG and connectivity, we repeated the combined analysis for each cognitive domain (memory, processing speed and executive function and attention). The results are summarized in Table 4. Lifestyle was associated with all cognitive domains when comparing those in the highest LIBRA risk tertile to the lowest (memory: *β* = 0.102, *P* = 0.001; processing speed: *β* = 0.086, *P* < 0.001; executive function and attention *β* = 0.109, *P* < 0.001). For memory, this association was partially mediated by BAG (*β* = −0.019, *P* < 0.001; proportion mediated (PM) = 15.6%), and by the combined pathway (*β* = −0.001, *P* < 0.001; PM = 1.2%), but not by connectivity alone, resulting in a total mediation of 17.7% (*β*_indirect_ = −0.022, *P* < 0.001; *β*_total_ = −0.124, *P* < 0.001). The association between lifestyle and processing speed was partially mediated by BAG (*β* = −0.024, *P* < 0.001; PM = 19.4%), connectivity (*β* = −0.008, *P* < 0.001; PM = 6.0%) and the combined pathway (*β* = −0.007, *P* < 0.001; PM = 5.9%), resulting in a total mediation of 31.2% (*β*_indirect_ = −0.039, *P* < 0.001; *β*_total_ = −0.125, *P* < 0.001). Finally, the association between lifestyle and executive function and attention was also partially mediated by BAG (*β*_indirect_ = −0.019, *P* < 0.001; PM = 14.4%), connectivity (*β*_indirect_ = −0.003, *P* < 0.001; PM = 2.5%) and the combined pathway (*β* = −0.003, *P* < 0.001; PM = 2.5%), resulting in a total mediation of 19.3% (*β*_indirect_ = −0.026, *P* < 0.001; *β*_total_ = −0.135, *P* < 0.001).

**Table 4:** Results of structural equation modelling by cognitive domain

	Combined cognition (*N* = 4881)	Memory (*N* = 4802)	Processing speed (*N* = 4772)	Executive function and attention (*N* = 4760)
**Direct effects**				
*on Brain-age gap*				
LIBRA tertile = 2	0.106, *P* < 0.001	0.107, *P* < 0.001	0.106, *P* < 0.001	0.107, *P* < 0.001
LIBRA tertile = 3	0.271, *P* < 0.001	0.269, *P* < 0.001	0.263, *P* < 0.001	0.263, *P* < 0.001
*on Connectivity*				
LIBRA tertile = 2	−0.007, *P* = 0.832	−0.005, *P* = 0.871	−0.006, *P* = 0.858	−0.007, *P* = 0.826
LIBRA tertile = 3	−0.108, *P* = 0.002	−0.103, *P* = 0.003	−0.109, *P* = 0.002	−0.108, *P* = 0.002
Brain-Age Gap	−0.412, *P* < 0.001	−0.408, *P* < 0.001	−0.411, *P* < 0.001	−0.411, *P* < 0.001
*on Cognitive performance*				
LIBRA tertile = 2	−0.088, *P* = 0.001	−0.106, *P* < 0.001	−0.044, *P* = 0.047	−0.036, *P* = 0.145
LIBRA tertile = 3	−0.151, *P* < 0.001	−0.102, *P* = 0.001	−0.086, *P* < 0.001	−0.109, *P* < 0.001
Brain-Age Gap	−0.126, *P* < 0.001	−0.072, *P* < 0.001	−0.092, *P* < 0.001	−0.074, *P* < 0.001
Connectivity	0.058, *P* < 0.001	0.013, *P* = 0.308	0.068, *P* < 0.001	0.031, *P* = 0.006
**Indirect effects**				
*via Brain-age gap on connectivity*				
LIBRA tertile = 2	−0.044, *P* < 0.001	−0.044, *P* < 0.001	−0.043, *P* < 0.001	−0.044, *P* < 0.001
LIBRA tertile = 3	−0.111, *P* < 0.001	−0.110, *P* < 0.001	−0.108, *P* < 0.001	−0.111, *P* < 0.001
*via Brain-age gap and connectivity (on cognitive performance)*				
LIBRA tertile = 2	−0.016, *P* < 0.001	−0.008, *P* = 0.001	−0.013, *P* = 0.001	−0.016, *P* < 0.001
LIBRA tertile = 3	−0.047, *P* < 0.001	−0.022, *P* < 0.001	−0.039, *P* < 0.001	−0.047, *P* < 0.001
Brain-age gap	−0.024, *P* < 0.001	−0.005, *P* = 0.308	−0.028, *P* < 0.001	−0.024, *P* < 0.001

Dependent variables in italics, with predictor variables listed below.

A dementia risk score ranging from −2.7 to 10.2, such that a higher score reflects a higher risk. LIBRA score was split into tertiles; associations presented here are in reference to the lowest-risk tertile (LIBRA tertile = 1). Tertile ranges: 1 = (−2.7–0); 2 = (0.1–1.9); 3 = (2.0–10.2).

LIBRA, LIfestyle for BRAin health.

### Sensitivity analysis

Due to the sampling strategy of The Maastricht Study, a relatively high number of individuals with T2DM were included in the analyses. To test whether the results found were driven by individuals with T2DM, we redid the analysis with diabetes status excluded from the LIBRA calculation, and controlled for separately in structural equation modelling. In this case, the associations between LIBRA and cognitive function weakened to some extent (*β*_original_ = −0.198, *P* < 0.001; *β*_modified_ = −0.135, *P* < 0.001), while the PM remained similar (∼21%). We additionally tested the interaction by T2DM status, and this was not significant (*χ*^2^ = 0.31, *P* = 0.580). Finally, interaction with sex was tested, and it was found that the association between lifestyle and BAG is stronger in men (*χ*^2^ = 13.12, *P* < 0.001).

## Discussion

In this study, we jointly tested the associations between lifestyle factors for brain health, BAG and connectivity on cognitive functioning in a large cohort of middle-aged community-dwelling adults. The results show that people with a healthier lifestyle (as summarized by the LIBRA score) had lower BAG, higher connectivity and better cognitive function. A lower BAG was an important mediator of these cognitive differences. Similarly, albeit to a lesser extent, better white matter connectivity mediated better cognitive function in those with healthy lifestyle.

The finding that lifestyle is related to lower BAG is supported by another recent study,^38^ which found in a sample of 938 participants at mid-life that higher LIBRA scores were associated with higher BAG, and by Chen *et al*.,^39^ who found in a sample size of 326 that BAG functions as a mediator for various lifestyle dementia risk factors, including education. The present study results provide evidence in a large population-based sample that mid- and late-life modifiable risk factors (while controlling for early-life education) are also associated with cognitive function via BAG. This suggests that lifestyle factors may be relevant for cognition throughout the life course, and not just at early ages.

This finding supports the cognitive reserve hypothesis, which states that engagement in brain-healthy lifestyle can reduce an individual’s susceptibility to cognitive decline.^40^ Cognitive resilience to age- and pathology-related brain changes is theorized to be imparted via separate pathways, namely, the preservation of brain structure (brain reserve) and the stimulation of brain activity (cognitive reserve), respectively. Previous findings have demonstrated that white matter connectivity underlies cognitive resilience, in a construct referred to as neural reserve.^41^ Here, we provide evidence that brain structure and white matter connectivity separately mediate associations between lifestyle and cognition in a manner predicted by the reserve hypothesis. The cognitive reserve aspect of the hypothesis is not fully captured in our model. However, this could be an explanation for the portion of the LIBRA–cognitive function association that was not mediated by either BAG or connectivity, i.e. the direct association between LIBRA and cognition.

In The Maastricht Study, modifiable dementia risk factors have previously been related to differences in markers of atrophy, cerebral small vessel disease and cognition.^7^ In the present study, we validated and extended this finding by showing that the associations with brain volumetric markers can be readily captured by calculating a single metric, BAG and that both lifestyle and BAG relate to structural connectivity, and thereby jointly explain differences in cognitive function.

The BAG model had an adjusted *R*^2^ of 0.447—indicating that almost half of the variance in the difference (gap) between calendar and brain ages was predicted by CSF and WMH volumes (*P* < 0.001). These factors represent global measures of atrophy and microvascular damage in the brain, respectively. This approach differs from that of previous BAG models, which most commonly assess only regional variation in GM, WM and CSF volumes.^42^ The combination of atrophic and vascular damage metrics may allow this model to better capture multifactorial brain damage. This BAG model performance (mean absolute error = 6.4 years) matches other research that has used similar methods (mean absolute error = 6.9 years),^43^ but does not achieve results that are as precise as that of popular machine or deep learning–based techniques, which achieved mean absolute error of 2.14 years.^44^ One advantage of our simpler, regression-based approach is that this technique is less computationally demanding and can be easily applied to datasets that only have whole-brain volumes available. Additionally, this method allows for relatively simple interpretation of which imaging features are most relevant to the model, in contrast to the ‘black box’ machine or deep-learning techniques.

Conversely, however, the residuals from our brain-age prediction model partly consist of model error that cannot be explained by lifestyle and other covariates in the model, which might have diluted the observed associations. It is possible that more precise brain-age measures would result in less model error, capture a greater portion of the variance in cognition attributable to lifestyle factors and reduce the direct association between lifestyle and cognition that is independent of brain structure (attributed to cognitive reserve). Brain connectivity has previously been shown to underlie this association,^41^ but did not strongly influence the brain-age measure in comparison with the contributions of vascular and atrophic damage markers. Age-related changes to connectivity networks are small and subtle relative to tissue loss and WMH load. It is probable, however, that a more complex analysis of the brain networks, especially exploring local connectivity features, would better quantify age-related changes to the structural connectivity network. Future work should incorporate more complex measures of the interplay between brain connectivity and structure.

The results of the cognitive domain-specific analyses support this idea as well. When we repeated the analysis separately for each cognitive domain, we found that both lifestyle and BAG were relevant to all three domains. However, connectivity measures were not associated with performance in the memory domain, but were significant for executive function and attention and processing speed. These findings are in line with previously published research,^45,46^ which have found that declines in executive function and attention and processing speed are associated with global alterations in structural connectivity, while memory performance is associated primarily with connectivity local to the hippocampus. This finding supports the idea that executive function and attention and processing speed involve more distributed networks in the brain, and hence are more readily detected with global connectivity measures. Future work should also explore local network alterations to better assess associations of structural connectivity with memory performance.

### Study considerations

This analysis has some important strengths. Notably, the results were found in a large, community-dwelling sample, which suggests that lifestyle impacts on brain ageing are relevant to the general population. Furthermore, the deep phenotyping approach of the study enables comprehensive modelling of pathways in the reserve theory. Additionally, the use of whole-brain volumes and a relatively simple, explainable linear regression approach to brain-age modelling allows this analysis to be readily replicated in other datasets. Finally, the combination of several comprehensive measures into composite scores for cognition and lifestyle increase the robustness of the analysis and reduce the likelihood of Type 1 error.

There are also some limitations. The analyses were based on cross-sectional data, which limits our ability to explore causal pathways, or rules out the possibility of reverse causation, such that worse cognition actually leads to unhealthier lifestyles. Furthermore, although sensitivity analyses showed no significant differences, the study population consists of an oversampling of individuals with type 2 diabetes, which may differ from the general population. Additionally, the whole-brain nature of the measures, while convenient in terms of availability in other studies and robustness, prevents detection of associations with more regional brain measures. These measures have been shown previously to predict brain age and may account for some additional mediation of brain reserve to cognition. Such reasoning suggests that the mediation effect found here is in fact an underestimate of the true effects.

## Conclusion

To conclude, associations between health- and lifestyle-based risk/protective factors (LIBRA) and cognition can be partially explained by structural brain health markers (BAG) and white matter connectivity markers. Lifestyle interventions targeted at high-risk individuals at any age may be effective in promoting and preserving cognitive function in the general public.

## Data Availability

Data from The Maastricht Study are available upon reasonable request for researchers who meet the criteria for access to confidential data; the corresponding author may be contacted to request data.
